# Tracking of Mesenchymal Stem Cells with Fluorescence Endomicroscopy Imaging in Radiotherapy-Induced Lung Injury

**DOI:** 10.1038/srep40748

**Published:** 2017-01-19

**Authors:** Jessica R. Perez, Norma Ybarra, Frederic Chagnon, Monica Serban, Sangkyu Lee, Jan Seuntjens, Olivier Lesur, Issam El Naqa

**Affiliations:** 1 McGill University, Biomedical Engineering, Montreal, H4A 3J1, Canada; 2 McGill University Health Center, Medical Physics, Montreal, H4A 3J1, Canada; 3 Sherbrooke University, Intensive Care Unit and Pulmonology, Sherbrooke, J1H 5N4, Canada; 4 University of Michigan, Radiation Oncology, Ann Arbor, MI 48103-4943, USA

## Abstract

Mesenchymal stem cells (MSCs) have potential for reducing inflammation and promoting organ repair. However, limitations in available techniques to track them and assess this potential for lung repair have hindered their applicability. In this work, we proposed, implemented and evaluated the use of fluorescence endomicroscopy as a novel imaging tool to track MSCs *in vivo*. MSCs were fluorescently labeled and injected into a rat model of radiation-induced lung injury via endotracheal (ET) or intravascular (IV) administration. Our results show that MSCs were visible in the lungs with fluorescence endomicroscopy. Moreover, we developed an automatic cell counting algorithm to quantify the number of detected cells in each condition. We observed a significantly higher number of detected cells in ET injection compared to IV and a slight increase in the mean number of detected cells in irradiated lungs compared to control, although the latter did not reach statistical significance. Fluorescence endomicroscopy imaging is a powerful new minimally invasive and translatable tool that can be used to track and quantify MSCs in the lungs and help assess their potential in organ repair.

Stem cell therapy has been proposed for many years to repair damaged organs. However, little clinical success has been achieved with a few exceptions, and stem cell therapy remains at the research stage. One of the hindering reasons is due to our lack of knowledge and understanding of the biological location and the mechanisms of action of stem cells after being injected.

Bone marrow derived stem cells, mesenchymal stem cells or marrow stromal cells (MSCs) have demonstrated great promise in regenerative medicine for multiple organs including the lungs^1^. Although MSCs therapy showed beneficial effects in the treatment of injured tissue, their mechanisms of action are still being investigated. In particular, rather than MSCs engrafting in the tissue and differentiating into lung tissue for repair, it appears now that this may be a fairly rare event and that the main beneficial effects of MSCs may lie in their paracrine immunomodulatory capacity^2^,^3^. Clinical trials are underway using MSCs for the treatment of inflammatory diseases, including chronic obstructive pulmonary disease (COPD), which relies only on anti-inflammatory properties of MSCs to improve lung function^2^.

Radiotherapy is used routinely as part of cancer treatment. Among patients undergoing radiation therapy, some will develop side effects, which could seriously impact their quality of life and lead to complications and even death^4^. The therapeutic window between delivering high enough dose to the cancer while still sparing normal tissue is critical in radiation oncology^5^. Radiation-induced lung injury (RILI) occurs in about 30% of thoracic irradiations and is one of the major limiting factors for increasing the dose to achieve better tumor control^6^. RILI is an inflammatory process, with the combination of damage to parenchymal cells as well as vascular endothelium and connective tissue, and including the release of cytokines and growth factors^4^. Currently, limited therapeutic options are available so MSCs immunomodulatory effects could potentially offer an alternative to mitigate those effects.

Assessing the potential of MSCs for tissue regeneration suffers from limitations in available techniques to follow the cells^7^. This is currently done by *in vitro* methods such as immunohistochemistry (IHC) and real-time polymerase chain reaction (RT-PCR). Those techniques, although very informative require the sample to be either fixed for IHC or destroyed for RT-PCR. A non-invasive method that would be able to track the cells *in vivo* continuously would hence be of great interest to investigate the regenerative potential of MSCs mediated tissue repair and to better understand the underlying dynamics of the process^8^.

With the advent of molecular imaging, a number of questions with previously unknown answers can now be addressed^9^. Whole body imaging techniques have been proposed to track delivered stem cells in an injury model, including positron emission tomography (PET), Magnetic resonance imaging (MRI) and optical imaging methods. PET gives 3D imaging and is highly sensitive, however it requires the use of a radioactive tracer and suffers from poor resolution. MRI gives high 3D resolution and great tissue contrast but is still an expensive and complicated method. Bioluminescence imaging is specific and simple but does not have 3D capabilities and is limited to surface imaging in preclinical models^9^. Fluorescence endomicroscopy is minimally-invasive and has the ability to image accessible organs such as the lungs at the cellular level and in real time^10^. This new technology is also amenable to fluorescence endomicroscopy and has been used to detect real-time cellular enzymatic activity (e.g. myeloperoxidase)^11^ or to follow fluorescently labeled cells *in vivo*^12^. MSCs have been imaged with endomicroscopy in a preclinical model of inflammatory bowel syndrome^13^.

In this work, we investigate the possibilities of using endomicroscopy in conjunction with fluorescently labeled MSCs to assess their behavior in a radiation-induced lung injury model. Endomicroscopy is a promising technique that is currently used clinically and therefore opens new doors in terms of the translational potential of this investigation.

The study presents a multidisciplinary effort that combines: (1) The establishment of a rat model for imaging RILI that includes, to the best of our knowledge, first time investigation of endomicroscopy imaging techniques to assess such model. Our developed rat model mimics treatment planning and delivery procedures of radiation therapy that are consistent with current clinical practice to simplify its potential translation; (2) Application of MSCs therapy as an agent to mitigate radiation induced lung damage. This involves extraction of MSCs, culturing and optimization of cell labeling for real-time tracking purposes; (3) The optimization of an endomicroscopy imaging protocols for our application in MSCs tracking, which involved several pilot studies to ensure feasibility and efficacy (control vs injured rats, MSCs injection protocol both endotracheal and intravascular, and cell visualization); (4) The design, validation and application of quantitative image processing algorithms to customize automated cell counting from *in vivo* endomicroscopy video aquisitions for comparing different injection routes and imaging conditions.

## Results

### *In vivo* imaging of MSCs

In order to assess fluorescence endomicroscopy as an imaging tool for detection of MSCs, we labeled them in culture with membrane dye DiD prior to injection. We chose DiD for its ease of use and its low cell toxicity. Light tissue penetration and autofluorescence are indeed a recurring issue in optical imaging. In our case, we are interested in “surface” imaging of the lining epithelial tissue of the lungs and tracking MSCs fate in a lung injury model with endomicroscopy. Thus, the premise of this work, does not require deep tissue penetration. However, we are also limited by the available lasers and filters technologies in the microendoscope, which contains two channels: green (488 nm) and red (660 nm). Therefore, we chose DiD to match the red channel available since NIR dyes are not per se supported on such a microendoscope system, as applications are limited to surface imaging. On the other hand, 660 nm is not so far to the bottom line level of NIR channel (ie 700 nm). We also chose the red rather than the green to limit excess autofluorescence which is more prominent in the green region of the light spectrum.

To evaluate the impact of radiotherapy and the different routes of MSCs administration with imaging, rats were divided into 4 groups (n = 5 per group): control with intravascular injection of MSCs (Ctrl MSC-IV), control with endotracheal injection of MSCs (Ctrl MSC-ET), irradiated with intravascular injection of MSCs (RT MSC-IV) and irradiated with endotracheal injection of MSCs (RT MSC-ET). MSCs administration and endomicroscopy imaging were conducted 3 weeks post-radiation therapy to follow the distribution of labeled-MSCs.

**Figure 1:**
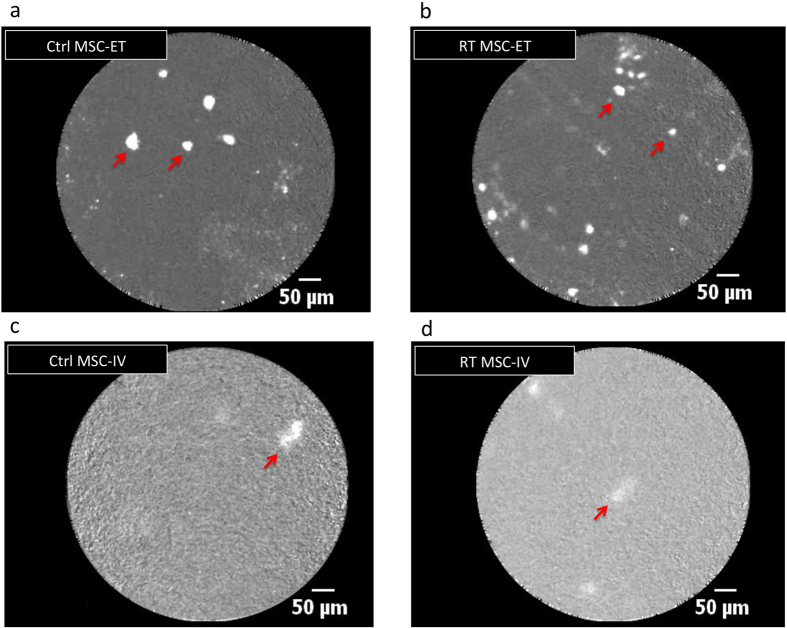
*In vivo* fluorescence endomicroscopy imaging of labeled MSCs in the lungs for each injection route and condition. Representative images from video sequences. MSC appear as bright spots (examples of cells shown by red arrows). (**a**) Ctrl-MSC ET. (**b**) RT-MSC ET. (**c**) Ctrl-MSC IV. (**d**) RT-MSC IV. Cells appear brighter and more numerous in ET injection (top row) compared to IV (bottom row). More cells are observed in irradiated lungs (right) compared to controls (left).

We were able to detect injected MSCs for both delivery routes, and in radiation damaged lungs. A sample video of MSCs injected ET in an irradiated rat model imaged with fluorescence endomicroscopy is available in supplementary material. Figure 1 shows representative *in vivo* fluorescence endomicroscopy images for endotracheal and intravascular injection of MSCs in control and irradiated lungs. Labeled MSCs appear as bright spots on the images. Qualitatively, there appear to be more MSCs in the ET group compared to IV.

### Image analysis and quantification

**Figure 2:**
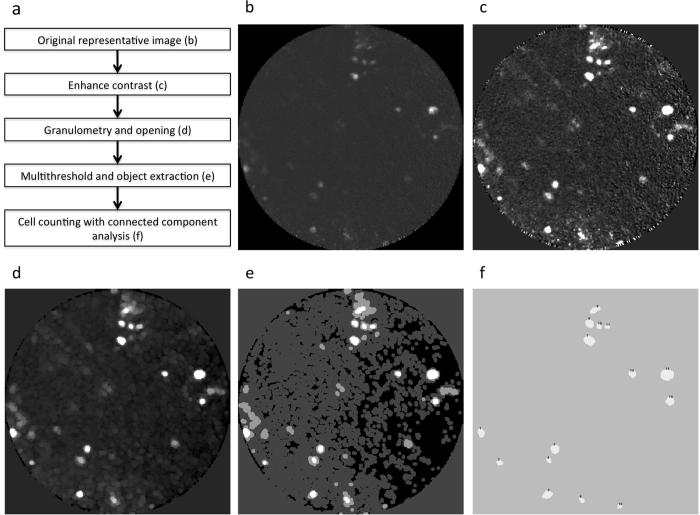
Automatic cell counting algorithm for image quantification. Each frame in the video sequence is treated as a stand alone image. (**a**) Summary of workflow. (**b**) Original representative image. (**c**) First contrast is enhanced followed by granulometry (**d**) to determine objects size and image opening. (**e**) Then, a threshold is applied to highlight bright cells. (**f**) Finally, cells are counted with connected component analysis.

In order to quantify the visual differences between conditions, we developed an automatic cell counting algorithm in Matlab. Each frame of the acquired video is treated as a stand alone image and objects (MSCs) are counted on each frame using a granulometry approach. Figure 2 describes the different steps of the automated cell counting algorithm.

#### Cell counting algorithm validation

**Figure 3:**
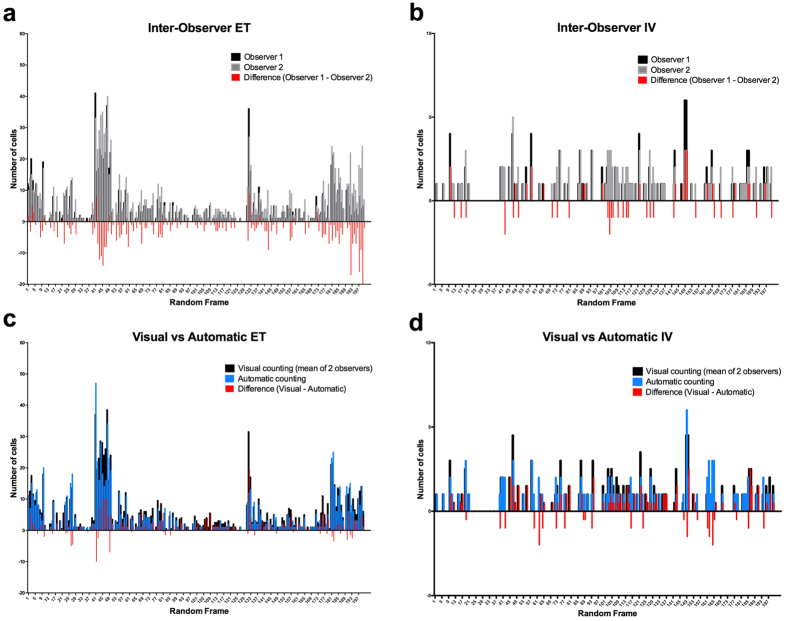
Automatic cell counting algorithm validation. Graph represents number of counted cells for each random frame comparing inter-observer variability and visual vs automatic counting with residual differences. (**a**) Inter-observer variability ET. (**b**) Inter-observer variability IV. (**c**) Visual vs Automatic ET. (**d**) Visual vs Automatic IV.

The developed cell counting algorithm was validated on 400 random video frames (200 for ET and 200 for IV, including control and RT). MSCs were counted on each random frame both visually (ground truth, average of 2 independent observers) and automatically (Fig. 3). We obtained a concordance correlation coefficient (CCC) of 0.91 (1 being perfect agreement) for ET and a CCC = 0.73 for IV. To assess inter-observer variability we compared cell counts from two independent observers and obtained a CCC = 0.85 for ET and CCC = 0.81 for IV.

#### Cell Counting in Video Sequence

**Figure 4:**
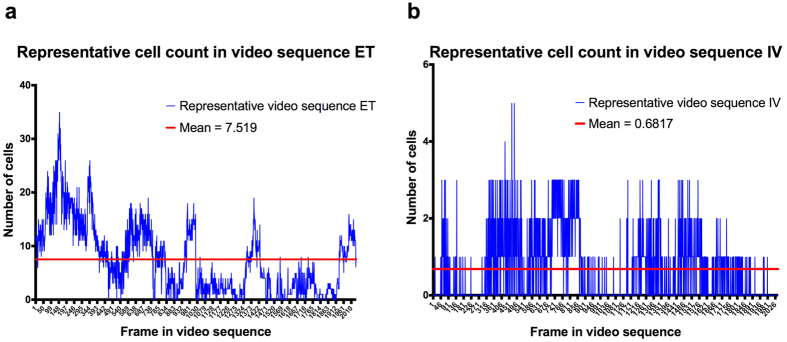
Number of detected cells per video frame in a representative video sequence. Representative cell count per frame using the automatic cell counting algorithm for ET (**a**) and IV (**b**). As the microendoscope probe moves in the lungs acquiring images, the number of detected cells varies.

Once the automatic cell counting algorithm was validated, we applied it to full video sequences. As the endoscope probe moves through the lungs acquiring images, we compute the number of detected cells per frame in each video sequence (Fig. 4). The number of detected cells varies depending on the region being imaged and motion, including breathing and heartbeat.

### MSCs imaging in different conditions

#### Impact of MSCs delivery routes

**Figure 5:**
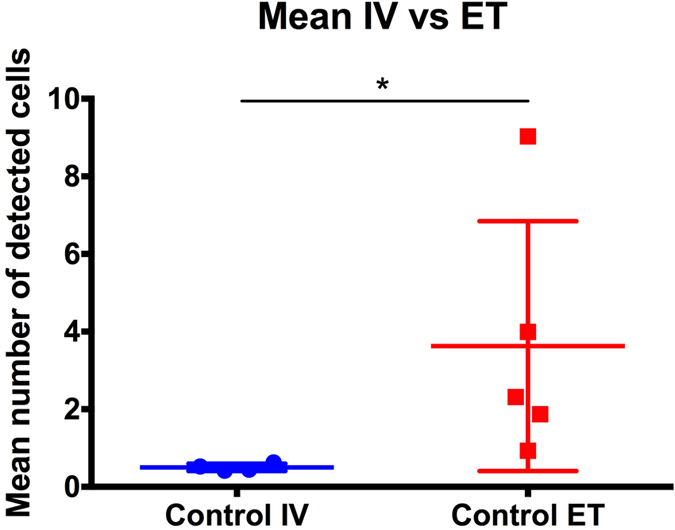
Mean number of detected cells for intravascular and endotracheal injection. The mean number of detected cells was computed for each video and each rat (each point is one rat with two videos per rat). The horizontal bars represent the mean and standard deviation. The mean number of detected cells differed significantly with 0.5 for IV and 3.6 for ET (p = 0.02).

We compared two MSCs delivery routes, ET or IV. Using the automatic cell counting algorithm, we computed the mean number of detected cells for both conditions in each rat (Fig. 5). We observe significantly more MSCs in ET than IV with a mean number of detected cells of 3.6 compared to 0.5, respectively (Mann-Whitney test with p = 0.02) (Table 1, control column).

#### Impact of RILI

**Figure 6:**
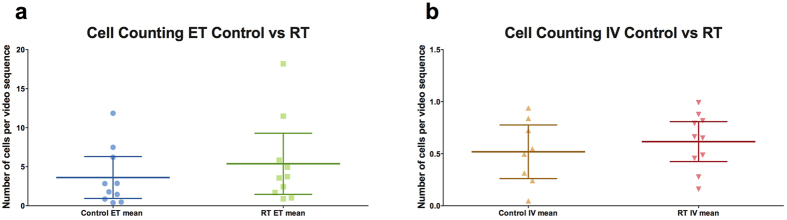
Mean number of detected cells in control and irradiated rats for intravascular and endotracheal injection. Each point represents a video (two videos per rat) and the horizontal bars represent the mean with 95% CI. A higher mean number of detected cells was observed in the radiation group for both ET with 3.6 for control and 5.4 for irradiated and IV with 0.5 for controls and 0.8 for irradiated. However, those differences were not statistically significant.

To evaluate the impact of radiation damage on MSCs imaging, we developed a rat model of RILI. We compared images from control rats and irradiated rats (RT) using the cell counting algorithm. We observed an increase in the mean number of detected cells in RT compared to control for both groups with 3.6 cells for controls and 5.4 for RT in the ET group, and 0.5 for controls and 0.8 for RT in the IV group (Fig. 6). However, these differences did not reach statistical significance (Mann-Whitney test with p = 0.39 for ET and p = 0.63 for IV). Table 1 summarizes the mean number of detected cells per condition.

We also computed the total number of cells for each video sequence. We obtain a mean total number of cells of 3698 for Ctrl MSC-ET and 5488 for RT MSC-ET, and 516.9 for ctrl MSC-IV and 612.4 for RT MSC-IV. The total number of cells also shows a higher number of cells in the RT groups, however these differences did not reach statistical significance either.

### Histology and Microscopy

**Figure 7:**
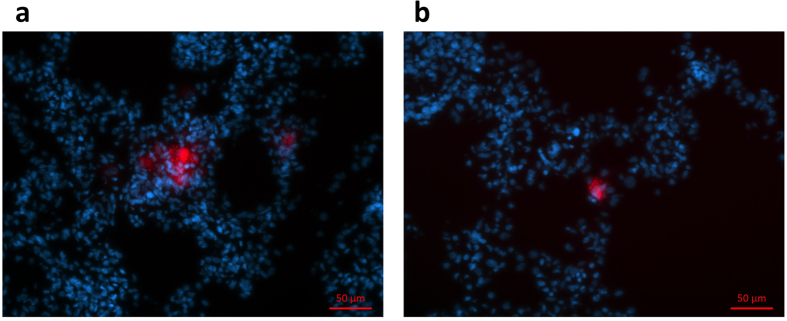
Fluorescence microscopy of lung sections post-endomicroscopy imaging. MSCs appear red stained for *in vivo* imaging with DiD. Nuclei stained blue with DAPI. (**a**) Lung sections of rat where MSCs were injected ET and (**b**) IV.

Following *in vivo* fluorescence endomicroscopy imaging, lungs were harvested, fixed frozen and sectioned. Lung sections were also stained for nuclei in the tissue. We were able to detect previously labeled and injected DiD-MSCs for both ET and IV delivery (Fig. 7). However, very few cells were detectable in lung sections.

## Discussion

We proposed and evaluated the use of fluorescence endomicroscopy imaging to detect MSCs in the lungs. We were able to detect fluorescently labeled MSCs injected both IV and ET as well as in a RILI model. We also developed an automated cell counting algorithm to calculate the mean number of cells detected in endomicroscopy video sequences.

The choice was made to use DiD as a labeling method for its ease of use and its low cell toxicity. DiD being a lipophylic dye, it is expected to remain confined into the lipid bilayer of the cell membrane, as has been demonstrated by previous studies^14^. However, it is possible for the dye to transfer from the labeled cell to an unlabeled adjacent cell if the membranes are in contact. The labeling becomes less intense as the dye is diluted each time the cell divides. Fluorescent debris might occur if the cell dies or the membrane breaks. Debris would appear smaller than cells and we implemented in our counting algorithm a size constraint as well as a roundness shape structure to take this issue into account. Debris of dead cells could also be taken up by macrophages and the labeling would be on macrophages instead of MSCs thus creating possible false positives. In our case, the experimental time frame was specifically chosen to mitigate such concerns; cells were labeled 2 days prior to injection and washed so that the excess dye outside the cells (background) was removed. Cells were injected and rats were immediately imaged with microendoscopy, so that DiD would not have sufficient time to be released from the cells by the time we imaged. After a few hours or days, the dye is released by the dying MSCs or MSCs fusing with other cells and then, specificity in the detected signal may be lost. However, the timing between injection and imaging was kept tight and no degradation of DiD was observed. Extensive testing of the stability of DiD labeling of MSC *in vivo* was deemed outside the scope of the present study. Nonetheless, the study was designed such that there is very little time between injection and imaging to avoid any possible loss of specificity of the detected signal, which was not observed in our studies. DiD was used by others to image cells 5 days after labeling and injection^14^. While we cannot rule out that some DiD did leak out of the membrane, the manufacturer of the dye clearly states that DiD does not readily transfer to unlabeled cells. While the dye could be taken in by phagocytes after MSC death but not necessarily re-integrated in membranes by other cells, the labeling in the cells would then be much lower or exhibiting distinctive pattern than that of MSC. We used a simple membrane dye to label MSCs, but this method is amenable to many different labeling solutions. For example, MSCs could be labeled with two different colors and/or with a reporter gene system. Cell labeling can also be combined with anatomical or functional markers to answer more advanced biologically relevant questions.

IV MSCs injection showed much fewer cells on the images compared to ET. Our goal is to track the administrated MSCs and monitor their interaction with the lung epithelial tissue, which is more likely to impact the ability of these cells to reverse radiation-induced lung injury. The fact that microendoscopy is a surface optical imaging helps with this regard. This explains why ET injected cells, which appear on the same side of the lungs as the endoscope probe could be tracked and imaged well. IV injected cells can be detected but to a limited extent since some may have extravasated to the airways but many remain in the capillaries deeper below the surface. It is likely that some may still after longer periods become visible but such waiting raises other problems in terms of dye dilution and it is outside the scope of the current work.

The lung being a large organ and the endoscope tip being somewhat rigid, we are limited in terms of the surface or the volume of the lung that we could cover. This is a common limitation of all microendoscopic devices, which need direct contact to surface tissues in order to perform resolution imaging. However, our goal is to localize the presence or absence of MSCs in areas of the lung with suspected damage. Other methods are more appropriate for whole organ imaging such as PET, however this would provide a macroscopic image of where the majority of cells are located if there is enough of them in close proximity to produce a detectable signal and it lacks the necessary resolution to address cellular level distribution. By using microendoscopy, we obtain a microscopic view of MSCs distribution within the organ at the cellular level. This allows us to quantify cells at microscopic resolution *in vivo*. More importantly, we are interested in the paracrine interaction between lung cells and injected MSCs. The interpretation of results that ET cells are likely to be more confined to the lung, then, a surface imaging method is sufficient to address the question at hand as such cells would be within the proximity of the endoscope probe. However, many IV injected cells may still remain in the capillaries of lungs and in the general circulation, which are less accessible by this method and also explain why they are less likely to have an impact in repairing lung injury as shown by our previous results.

We tested this method on a RILI model and on average we detected more MSCs in irradiated lungs compared to controls. Since the variability in detected cells number is so large between frames of a video sequence (for example, sometimes detecting no cell and sometimes 10), the standard deviation on the mean was too wide to reach statistical significance. The MSCs injection happening at the time of imaging shows that an increased number of cells in RT is probably not due to the homing effect. We hypothesize that because the lung was injured and the tissue is collapsing following irradiation, upon MSCs injection, more cells were visible as they were more confined in injured lungs compared to normal tissue.

We developed and validated an automatic cell counting algorithm which yields results consistent with visual counting with differences similar to those observed between observers. However, our image analysis was based on treating each video frame as a stand alone image and averaging over all the frames present in the video sequence. That means that double-counting of cells may occur in consecutive frames of the same region, for example. Due to the nature of the microendoscopy videos (see supplementary sample video), it is not possible to keep track of each cell individually. From one frame to the other, cells may appear and disappear and there is no current way of knowing if it is a new cell or a cell that is coming back after having skipped a few frames. Due to respiratory motion and heartbeat, the cell count could be varying between adjacent or subsequent frames. Therefore, the average number of cells seem to represent the best estimate to quantify the cell count in these videos.

One of the drawbacks of this technique is the lack of a positional device tracking system which could provide more information on which part of the lung is being imaged at which time. When looking at the images alone we are unable to distinguish the location within the lung with accuracy. An improved deployment of such technology could consist of image-guided endomicroscopy to determine the location of the probe, which can then be correlated with fluorescence images acquired in real time.

We aimed to explore the potential of fluorescence endomicroscopy as a novel method to track MSCs *in vivo* for repair of radiation-induced lung injury. To the best of our knowledge, this is the first application of endomicroscopy for live tracking of MSCs in such lung injury model. Moreover, the method allows for real-time detection of administered cells *in vivo* with cellular resolution and can be used for studies of cell tracking in other injury models as well. We acknowledge the limitation characteristics of any optical-based imaging technique; however, there is significant value in the results presented here in that it demonstrates the feasibility of using endomicroscopy in accessible organs, such as the lungs, to help resolve current questions pertained to stem cell lung treatment, including the efficacy of different administration routes. Therefore, our study results show great promise for further developing this technology with potentially other cell types or different injury models.

In conclusion, fluorescence endomicroscopy is a powerful new technique to follow stem cells in damaged lungs but more work is still needed to increase its potential for stem cell therapy imaging.

## Methods

All experiments were approved by the Animal Care Committee at the Research Institute of the McGill University Health Centre and in accordance with the ethical guidelines of the Canadian Council on Animal Care.

### MSCs Isolation and Culture

MSCs were isolated as previously described^15^. Briefly, femurs of female Sprague-Dawley rats were harvested. Bone marrow was flushed through the bone, filtered (70 *μ*m) and pelleted by centrifugation at 300 g for 5 minutes. Cells were counted (cell counter) and plated in T75 flasks at a density of 500,000 cells per cm^2^. MSCs were cultured in Mesenchymal Stem Cell Growth Medium (MSCGM, Lonza, Switzerland) supplemented with antibiotic-antimycotic (Invitrogen, Thermo Fisher Scientific, USA). Media was changed after 24 h to select for adherent cells and subsequently every 3 days until 80% confluence was reached. MSCs were expanded and passaged as needed, never exceeding passage 4 for injection to rats.

### MSCs Labeling

DiD (Vybrant DiD Cell-Labeling Solution, Thermo Fisher Scientific, USA) was chosen to match the red channel (660 nm) available on the endomicroscope with absorption peak of 650 nm and emission of 670 nm. DiD was mixed with MSCBM media at a concentration of 10 *μ*M and added to MSCs in culture. MSCs were incubated with DiD-media for 30 minutes at 37 °C in 5% CO_2_. Following incubation, MSCs were washed twice with 1X sterile DPBS and fresh media was added. The next day, MSCs were checked for proper labeling with fluorescence imaging. Previous *in vitro* tests demonstrated no effect on cell survival when MSCs were labeled with DiD.

### MSCs Administration

For intravascular injection: 1 million DiD-labeled MSCs were resuspended in 1 mL of sterile DPBS and injected through canulation of the jugular vein at slow speed to prevent embolism. For endotracheal injection: 200,000 cells were resuspended in 0.2 mL of sterile DPBS and directly administered in the trachea through tracheotomy.

### Tracheotomy

Tracheotomy in laboratory rats has been described before^16^. All surgical procedures were performed using sterile technique and under intramuscular anesthesia with ketamine/xylazine. Rats were immobilized in supine position on the surgical table. A midline cervical skin incision was done, and the cervical trachea was exposed by vertical separation of the muscles. A 14G catheter was passed through the trachea to allow the endoscope probe to pass.

### Fluorescence Endomicroscopy Imaging

All imaging procedures were conducted with rats under intramuscular anesthesia with ketamine/xylazine. The fiber optic probe of the endomicroscope (Cellvizio dual band, Mauna Kea Technologies, France) was inserted through tracheotomy into the airways. Images were acquired in the red channel (660 nm) immediately post injection with 2 consecutive acquisitions of 2 minutes length for each animal. This short time frame aims to ensure that DiD is still confined to the lipid bilayer and hence, to reduce possible loss in detected signal specificity. Due to bronchial architecture and operator expertise the probe was most likely to be directed to the right lung where RT damage was present.

### Rat Model of RILI

A rat model for radiation-induced lung injury was established. Sprague-Dawley female rats were anesthetized with isoflurane and were imaged on a computed tomography (CT) simulation scanner (Philips Brilliance Big Bore, Philips Medical Systems, Bothell, WA, USA) following an optimized small animal protocol (120 kVp X-ray tube voltage, 175 mA tube current, 0.37 mm in-plane resolution, 0.4 mm axial resolution). To this end, the animals were placed in a prone position on an in-house built Styrofoam holder with reference markers for positioning reproducibility. The lungs, heart and spinal cord were contoured on the CT images. A single fraction of 18 Gy was prescribed to the right lung with a 6 MV photon beam. A hemithorax parallel-opposed 3D conformal treatment plan was designed (Eclipse™V 11.0, Varian Medical Systems, Palo Alto, California, USA) for each individual animal based on the CT image. Each plan was adapted to animal’s anatomy as shown in the CT image to avoid a toxic level of radiation to the spinal cord, heart and left lung. The prescribed dose was delivered using a clinical Novalis Tx linear accelerator (Varian Medical Systems, Palo Alto, California, USA). Anesthetized rats were positioned relative to the markers established at the planning CT. For each rat prior to irradiation, final positioning accuracy was established using cone beam CT.

### Image Analysis and Quantification

Regarding video sequence analysis, we treated each frame individually as a stand-alone image. Images were analyzed with Matlab (The Mathworks, Inc., USA). We first enhanced the contrast of the image. Then we applied a granulometry algorithm (as previously described in the Matlab image processing toolbox example “granulometry of snowflakes”) to our images in order to determine the size and number of objects (cells) present in the image without actually detecting individual objects first. To extract the cells, we opened the image with a disk structuring element of radius determined previously to obtain the granulometry image. A double threshold was subsequently applied to the resulting image to select fluorescent cells over background and the image was segmented in black and white. Using connected components analysis each detected object was counted.

In order to validate, this automatic cell counting method, we randomly selected 200 frames for the IV group and 200 for the ET group. Cells were visually counted on all random frames by two independent observers and the average of the two was used as ground truth for validation. The automatic cell counting algorithm was applied on the same frames and the concordance correlation coefficient (CCC) was computed.

Following validation the automatic cell counting algorithm was applied to all video sequences yielding cell number per frame for each video. The average cell count was extracted for each video and each rat.

### Histology and Microscopy

Following the imaging session, rats were euthanized and lungs were harvested. They were frozen in OCT (Tissue-Tek™O.C.T. Compound, Electron Microscopy Sciences, USA) and stored at −80 °C shielded from light. Lung sections were cut and stained with DAPI ((4′,6-Diamidino-2-Phenylindole, Dihydrochloride), ThermoFisher Scientific, USA) in order to detect cell nuclei and fluorescent DiD-MSCs. Images were acquired on a fluorescence microscope (AxioVert A1, Zeiss, Germany) with filters mPlum for DiD and DAPI and magnification 20X.

## Additional Information

**How to cite this article**: Perez, J. R. *et al*. Tracking of Mesenchymal Stem Cells with Fluorescence Endomicroscopy Imaging in Radiotherapy-Induced Lung Injury. *Sci. Rep.* **7**, 40748; doi: 10.1038/srep40748 (2017).

**Publisher's note:** Springer Nature remains neutral with regard to jurisdictional claims in published maps and institutional affiliations.

## Supplementary Material

Supplementary Video

Supplementary Material

## Figures and Tables

**Table 1:** Mean number of detected MSCs calculated with the automatic cell counting algorithm for each condition.

Mean number of detected MSCs	Control	Irradiated (RT)
Endotracheal (ET)	3.6	5.4
Intravascular (IV)	0.5	0.8
