# Supplementary material for: Tracking of Mesenchymal Stem Cells with Fluorescence Endomicroscopy Imaging in Radiotherapy-Induced Lung Injury
Source: Sci Rep. 2017 Jan 19;7:40748. doi: 10.1038/srep40748 (PMC5244404; doi:10.1038/srep40748)
Supplement: Supplementary Material [file srep40748-s2.pdf]

Tracking of Mesenchymal Stem Cells with Fluorescence Endomicroscopy Imaging in Radiotherapy-Induced Lung Injury

Jessica R. Perez, Norma Ybarra, Frederic Chagnon, Monica Serban, Sangkyu Lee, Jan Seuntjens, Olivier Lesur and Issam El Naqa

Supplementary material

Supplementary Video RT MSC-ET

Representative *in vivo* fluorescence endomicroscopy video sequence in irradiated lungs with MSCs injected ET.
